# The Practice and Potential Role of HIV Self-testing in China: Systematic Review and Meta-analysis

**DOI:** 10.2196/41125

**Published:** 2022-12-02

**Authors:** Xiangfei Xiu, Yuanyuan Qin, Yugang Bao, Yaokai Chen, Hao Wu, Xiaojie Huang, Lu Wang

**Affiliations:** 1 National Center for AIDS/STD Control and Prevention Chinese Center for Disease Control and Prevention Beijing China; 2 Clinical and Research Center for Infectious Diseases Beijing Youan Hospital Capital Medical University Beijing China; 3 Division of Infectious Diseases Chongqing Public Health Medical Center Chongqing China; 4 AIDS Healthcare Foundation Beijing China

**Keywords:** HIV, self-testing, China, meta-analysis, prevalence

## Abstract

**Background:**

HIV self-testing (HIVST) is recommended by the World Health Organization as a valid approach to routine HIV testing services. The scale of HIVST use has gradually been expanded in China over the past 5 years. To take a closer look at the role of HIVST in China, we reviewed the promotion and application of HIVST within China.

**Objective:**

The main objective of this study was to systematically analyze the proportion of past use and actual uptake of HIVST within China. Moreover, we aimed to quantify the effect of HIVST on HIV prevention and treatment.

**Methods:**

In all, 5 medical databases and 2 registration systems, including PubMed, Web of Science, MEDLINE, WanFang, China National Knowledge Internet, ClinicalTrials.gov, and the Chinese Clinical Trial Registry were systematically searched for studies reporting the prevalence of HIVST use from January 1, 2010, to December 25, 2021. Meta-analyses of the pooled proportion estimates were carried out by the meta-package in R software (version 4.1.2). Statistical heterogeneity among the studies was estimated using Cochran Q test and the inconsistency index (*I*^2^).

**Results:**

A total of 50 studies were included in our systematic review. The estimated pooled prevalence of HIVST use in China was 29.9% (95% CI 22.5%-37.9%). Among individuals who have ever used HIVST, 47.5% (95% CI 37.2%-57.8%) were tested for HIV for the first time. The pooled reactive rate of HIVST was 4.2% (95% CI 3.1%-5.8%). When HIVST revealed a reactive result, 81.3% (95% CI 70.9%-91.6%) of individuals sought medical care.

**Conclusions:**

In recent times, HIVST has become a valuable tool for HIV prevention in China. The widespread use of HIVST in non–men who have sex with men populations needs to be endorsed and promoted. The long-term applications of HIVST and the potential consequences of self-financing of HIVST in China have yet to be explored.

**Trial Registration:**

PROSPERO CRD42022304846; https://tinyurl.com/54d9pxy8

## Introduction

Approximately 5.9 million people were unaware that they were living with HIV in 2021, according to preliminary the Joint United Nations Programme on HIV/AIDS 2021 epidemiological estimates [1]. To achieve the goal of “95-95-95” as defined by the Joint United Nations Programme on HIV/AIDS, much work remains to be done, especially during the currently prevailing period when HIV services are being severely disrupted by the impact of COVID-19; in a worst case scenario, 7.7 million HIV-related deaths might possibly be incurred [2]. HIV self-testing (HIVST) has been shown to be an effective tool to potentially supplement other testing modalities to reach the 95-95-95 targets advocated by the World Health Organization [3].

HIVST has been recommended by the World Health Organization as a viable and effective extension to routine HIV-testing services since 2016. It has been shown to be a reliable strategy to promote HIV testing and simultaneously protects the privacy of those tested. Those tested can be made aware of their HIV infection status promptly by simply interpreting the test results, using the specimen collected by themselves at their convenience [4]. Oral fluid–based and blood-based HIVST are both accurate and practicable testing approaches in the study setting [5]. Additionally, the first urine-based HIVST testing kit was approved for use in China in 2019 [6]. Different categories of HIVST testing kits may mediate the acceptance and expansion of the use of HIVST within health care facilities. Previous meta-analyses have investigated the effects of HIVST and its purported benefits in key populations and have shown that HIVST plays a key role in HIV prevention by increasing the frequency of testing [7-9]. However, critical post-HIVST patient support needs to be diligently studied and holistically understood and includes issues related to the confirmation of results and linkage to ongoing care [7].

The scale of HIVST use has been expanded gradually within China [10]. The Chinese government released official directives encouraging the use of HIVST during the “Thirteenth Five-Year Plan” (2017-2022) period [11]. Since then, China has taken a series of measures to promote the expansion of HIVST. The National Center for AIDS/STD Control and Prevention and the Chinese Center for Disease Control and Prevention have conducted pilot HIVST projects in many cities across China and has worked with community-based organizations to explore more effective HIV-testing strategies [11]. Additionally, the HIVST strategy has been included in the National Guideline for Detection of HIV/AIDS, published by the Chinese Center for Disease Control and Prevention, since 2020 [12]. However, the expansion of the HIVST strategy in China has created both opportunities and challenges. The standardization, implementation, sustainability, and linkage of HIVST are concerns that have regrettably persisted [11].

Robust data, and the scrupulous analysis thereof, are required to define the practice and role of HIVST in China over the past decade, which may provide critical evidence for future decision-making related to HIVST promotion and application. Despite the existence of studies that have reported on the prevalent rates of HIVST in different areas or regions within China, the estimated HIVST prevalence rate at the national level has rarely been formulated [13-16]. To evaluate the practicality and influence of HIVST on HIV prevention in China, we conducted a systematic review and meta-analysis of studies that tracked the history and overall influence of HIVST on HIV prevention and treatment.

## Methods

The present systematic review and meta-analysis followed PRISMA (Preferred Reporting Items for Systematic Reviews and Meta-Analyses) guidelines. This study was duly registered with the International Prospective Register of Systematic Reviews (PROSPERO; registration number: CRD42022304846).

### Search Strategy and Selection Criteria

We searched for HIVST-associated studies that were conducted in China and published from January 1, 2010, to December 25, 2021, in 5 databases and 2 registration systems (PubMed, Web of Science, MEDLINE, WanFang, China National Knowledge Internet, ClinicalTrials.gov, and the Chinese Clinical Trial Registry) and then further reviewed them. Our search syntax was based on the following core concepts: “HIV,” “self-testing,” and “China” (see Multimedia Appendix 1 for details regarding the search strategy).

To qualify for inclusion, a research article had to meet the following criteria: (1) peer-reviewed articles reporting on the performance of rapid diagnostic HIV tests by those who self-tested, and (2) available numerator and denominator data to confirm rate values. Studies investigating foreign residents living within China were excluded.

### Selection Process and Data Extraction

Two reviewers (XX and YQ) independently screened and assessed the titles and abstracts of all articles identified via the search strategy. Each reviewer decided whether to exclude or include a study in accordance with a standardized form that defined the criteria for inclusion and exclusion. Data were independently extracted from individual studies by 3 reviewers (XX, YQ, and YB). If a dispute occurred, consensus was achieved through discussions with 3 other authors (HW, XH, and LW). EndNote reference management software (version X9.3.3; Clarivate) was used to filter duplicate studies. Microsoft Excel spreadsheet software (Microsoft Office 2016) was used to record the data extracted from eligible studies.

### Data Analysis

In all, 6 indicators were analyzed in this work: proportion of those who previously self-tested, proportion of actual uptake of HIVST, proportion of self-testing as lifetime first HIV screening, proportion of results feedback, reactive rate of HIVST, and proportion of linkage to care. The proportion of those who previously self-tested was defined as the number of individuals who have personal experience of HIVST among all the investigated people. The proportion of self-testing as lifetime first HIV screening was defined as the number of individuals who used self-testing as their first-ever HIV screening among people who have ever used HIVST. The proportion of results feedback was defined as the number of individuals who self-reported the results of HIVST or returned the images of HIVST to investigators or health institutions among those who self-tested for HIV. The reactive rate of HIVST was defined as the number of individuals receiving a positive reactive HIVST result among those who have self-tested for HIV. The proportion of linkage to care was defined as the number of patients who sought in-person confirmation of their HIV status (with or without accessing treatment) at local health facilities among individuals whose HIVST was reactive.

Meta-analyses of the pooled proportion estimates were carried out by the meta-package in R software (version 4.1.2; R Foundation for Statistical Computing). Statistical heterogeneity among the studies was estimated using Cochran Q test and the inconsistency index (*I*^2^). Very low, low, moderate, and high degrees of heterogeneity were defined as *I*^2^ of ≤25%, 25% to ≤50%, 50% to ≤75%, and ≥75%, respectively.

### Quality Assessment

The tool developed by Hoy et al [17] was used to assess the quality of the included studies [18]. The quality of each study was assessed according to 10 items with a maximum score of 10 (one point for a “Yes”). A total score of 0-5, 6-8, and 9-10 was considered high, moderate, and low risk of bias, respectively.

## Results

### Study Characteristics and Quality Assessment

**Figure 1:**
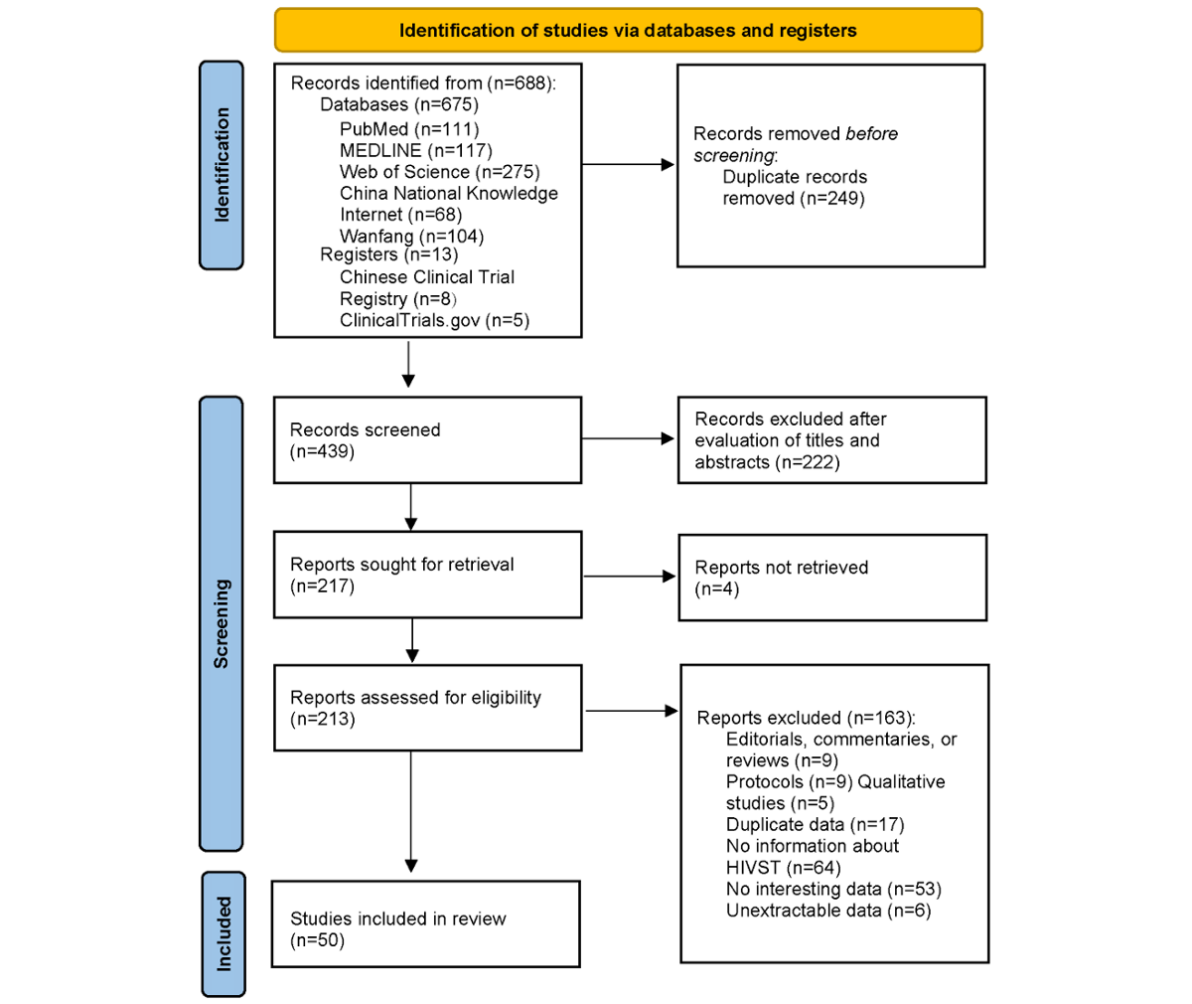
Flowchart presenting the selection of studies for inclusion in the systematic review and meta-analysis. HIVST: HIV self-testing.

A total of 688 records was found based on the initial search, of which 213 were selected for full-text evaluation after the removal of duplicates and initial screening. Finally, 50 studies were included in the systematic review based on critical appraisal and were included in our systematic review (Figure 1). Among them, 30 articles were written in the English language, and 20 articles were published in Chinese-language journals. Of the 50 studies, 6 (12%) were found to show a low risk of bias, 36 (72%) were classified as having a moderate risk, and 8 (16%) were classified as having a high risk of bias. The characteristics of eligible studies are summarized in Table 1.

**Table 1:** Characteristics of eligible studies.

Author	Publication year	Project year	Study design	Population	Setting	Provides HIVST^a^ kits	Fee for testing kits	Ways of supply	Quality score
Qi [19]	2013	2011-2012	Cohort	MSM^b^ with HIV-negative status	Beijing	Yes	Free	On-site	8
Han et al [20]	2014	2013	Cross-sectional	MSM	Guangdong and Chongqing	No	N/A^c^	N/A	7
Tao et al [21]	2014	2012	Cross-sectional	MSM with HIV-negative or unknown status	National	Yes	US $10 deposit	Postal	7
Wong et al [13]	2015	2013	Cross-sectional	MSM	Hong Kong	No	N/A	N/A	8
Yan et al [14]	2015	2013-2014	Cross-sectional	MSM with HIV uninfected or unknown status	Jiangsu	No	N/A	N/A	8
Zhong et al [22]	2017	2015	Cross-sectional	MSM	Guangdong	Yes	US $23 deposit	Postal	4
Qin et al [23]	2017	2015	Cross-sectional	MSM	National	No	N/A	N/A	9
Ren et al [24]	2017	2016	Cross-sectional	MSM with HIV-negative or unknown status having ever taken an HIV self-test	Beijing	No	N/A	N/A	8
Ren et al [15]	2017	2016	Cross-sectional	MSM with HIV-negative or unknown status	Beijing	No	N/A	N/A	7
Zhou et al [25]	2017	2016-2017	Cross-sectional	MSM	Guangdong	Yes	¥100 (US $13.94) deposit	Postal	6
Jin et al [26]	2017	2015-2016	Cross-sectional	MSM never tested for HIV	Unknown	Yes	Free	Postal	6
Jin [27]	2017	2016	Cross-sectional	MSM with HIV-positive status	Guangdong	No	N/A	N/A	5
Tang et al [28]	2018	2016	Cross-sectional	MSM	Guangdong and Shandong	No	N/A	N/A	9
Tang et al [29]	2018	2016-2017	RCT^d^	MSM HIV-negative or unknown status	Guangdong or Shandong	Yes	Free	Postal	8
Wei et al [30]	2018	2017	Qualitative participant observation study	MSM who self-report being HIV negative or unknown status	Jiangsu	Yes	Free	On-site	6
Wei et al [31]	2018	2017	Cross-sectional	MSM with HIV-negative status	Jiangsu	No	N/A	N/A	8
Xue [32]	2018	Unknown	Cross-sectional	MSM who self-report being HIV negative or unknown status	Guangdong and Shandong	No	N/A	N/A	4
Cheng et al [33]	2019	Unknown	RCT	Unknown	Unknown	Unknown	Unknown	Unknown	6
Fan et al [34]	2019	2017-2018	Cross-sectional	Students at 5 universities	Sichuan	Yes	Unknown	Postal or on-site	6
Tang et al [35]	2019	2016-2017	Cohort	MSM who self-report being HIV negative or unknown status	Guangdong and Shandong	No	N/A	N/A	8
Wei et al [36]	2019	2015-2017	Cross-sectional	MSM	Guangdong	No	N/A	N/A	9
Jin et al [37]	2019	2017	Cross-sectional	MSM with HIV-negative or unknown status	14 provinces in China	Yes	A US $5 deposit and a nonrefundable shipping fee of US $2 to US $3	Postal	6
Yang et al [38]	2019	Unknown	Cross-sectional	MSM who have ever tested for HIV	Nationwide	No	N/A	N/A	5
Zhu [39]	2019	Unknown	RCT	HIV-negative MSM	Anhui	Yes	Free	Postal or on-site	7
Mao [40]	2019	2017-2018	Cross-sectional	Unknown	Unknown	Yes	¥50 (US $6.97) deposit	Unknown	8
Zhao [41]	2019	2018-2019	Cohort	HIV-negative MSM	Anhui	Yes	Free	On-site	6
Liu et al [16]	2020	2017	Cross-sectional	MSM with HIV uninfected or unknown status	Chongqing	Yes	Free	On-site	9
Luo et al [42]	2020	2014-2016	Cross-sectional	Traceable sexual partners of newly diagnosed HIV-positive MSM	Zhejiang	Yes	Free	Sexual partners	7
Wang et al [43]	2020	2019	Cross-sectional	Female sex workers	7 provinces in China	No	N/A	N/A	9
Zhang et al [44]	2020	2018	Cross-sectional	MSM	National	Yes	Free	Postal or on-site	7
Luo et al [45]	2020	2019	Cross-sectional	MSM with HIV-negative or unknown status	National	No	N/A	N/A	7
Zhao [46]	2020	2017-2019	Cross-sectional	MSM	National	Yes	¥50 (US $6.97) deposit	Postal	7
Huang et al [47]	2020	2019	Cross-sectional	MSM	Guangdong	Yes	Unknown	Postal	7
Chan et al [48]	2021	2017	Cross-sectional	MSM	Hong Kong	Yes	Free	Postal or on-site	6
Cheng et al [49]	2021	2016	RCT	HIV-negative MSM	Guangdong	No	N/A	N/A	8
Hong et al [50]	2021	2019	Cross-sectional	MSM	Zhejiang	No	N/A	N/A	8
Lau et al [51]	2021	Before 2018 (unknown)	Cross-sectional	Male clients of female sex workers	Hong Kong	No	N/A	N/A	7
Li et al [52]	2021	2020	Cross-sectional	MSM who have ever tested for HIV	Jiangsu	No	N/A	N/A	7
Li et al [53]	2021	2017-2019	Cross-sectional	MSM	National	Yes	US $7 deposit	Postal or partner distribution	8
Ni et al [54]	2021	2019-2020	RCT	MSM	Unknown	Yes	US $15 deposit	Postal or partner distribution	3
Wu et al [55]	2021	2018-2019	Cross-sectional	MSM	Guangdong	Yes	US $15 deposit	Postal or partner distribution	6
Shan et al [56]	2021	2019	Cross-sectional	MSM	Beijing	No	N/A	N/A	5
Wu et al [57]	2021	2013,2014,2015, 2016,2018	Cross-sectional	MSM	National	No	N/A	N/A	3
Zhou et al [58]	2021	2017-2019	Cross-sectional	MSM	Guangdong	Yes	¥100 (US $13.94) deposit	Postal or partner distribution	6
Li et al [59]	2021	2020	Cross-sectional	MSM	Beijing	Yes	Unknown	Postal	6
Chu [60]	2021	2018	RCT	HIV-negative MSM	National	Yes	Free	Postal	5
Zhao et al [61]	2021	2019	RCT	MSM without syphilis or unknown status	National	Yes	Free	Postal	5
Jin et al [62]	2021	2018-2019	Cross-sectional	MSM with HIV-negative status	4 provinces in China	No	N/A	N/A	6
Bao et al [63]	2021	2018-2019	Cross-sectional	MSM who self-report being HIV negative or unknown status	Shanghai	No	N/A	N/A	6
Bao et al [64]	2021	2019-2020	Cross-sectional	MSM who self-report being HIV negative or unknown status	Shanghai	No	N/A	N/A	6

^a^HIVST: HIV self-testing.

^b^MSM: men who have sex with men.

^c^N/A: not applicable.

^d^RCT: randomized controlled trial.

### Proportion of Previously Self-tested

**Figure 2:**
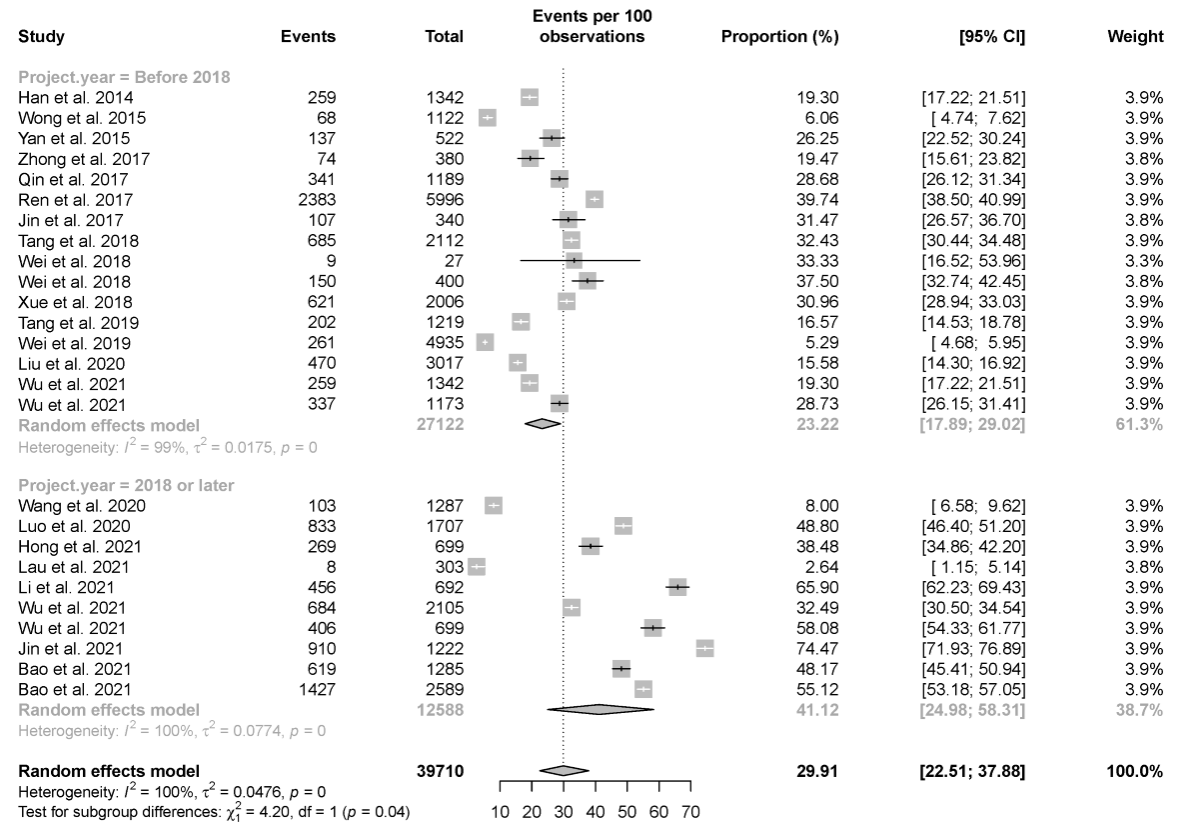
Pooled proportion of HIV self-testing use. Forest plot shows the estimated proportion of previously self-tested individuals before or after 2018.

A total of 23 studies investigated the proportion of those who previously self-tested. Only 2 studies reported on the prevalence of HIVST used in the past in HIV high-risk populations other than men who have sex with men (MSM) [43,51], with the lowest proportion of those who previously self-tested being the male clients of female sex workers (2.6%), as reported by Lau et al [51]. The highest prevalence of HIVST use in the past was observed in the study by Jin et al [62], that is, 74.5% in HIV-negative MSM. The estimated pooled prevalence of HIVST use in China was 29.91% (95% CI 22.51%-37.88%), with a higher estimated pooled prevalence in 2018 or later (41.12%, 95% CI 24.98%-58.31%) than before 2018 (23.22%, 95% CI 17.89%-29.02%; Figure 2).

### Actual Uptake of HIVST

**Figure 3:**
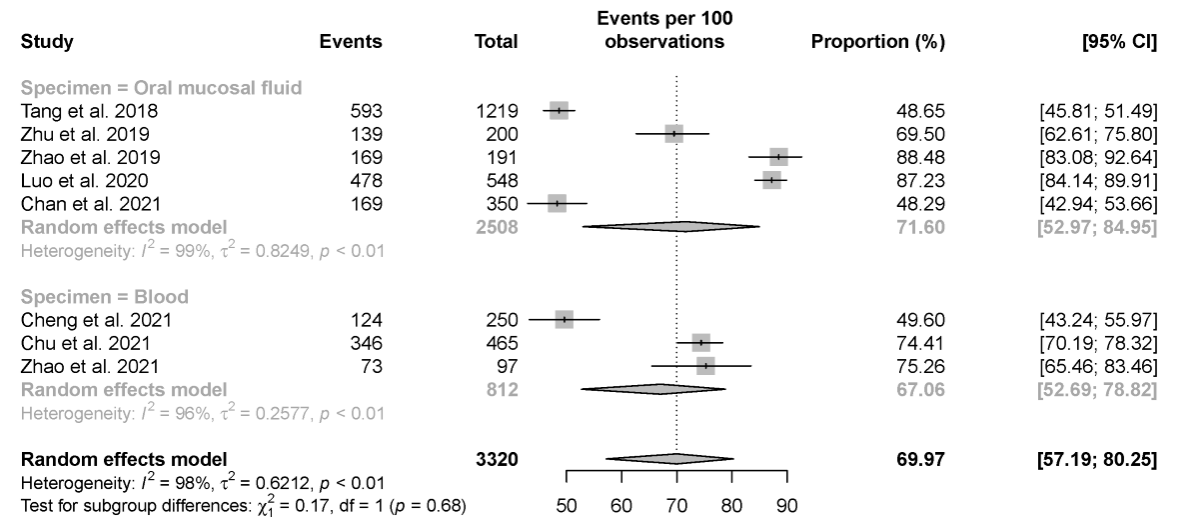
Pooled proportion of actual HIV self-testing uptake. Forest plot shows the estimated proportion of actual HIV self-testing uptake.

In all, 8 studies investigated the proportion of HIVST uptake after distributing HIVST kits to their study populations. The proportion of actual uptake of HIVST ranged from 48.29% to 88.48% across studies. It was shown that the pooled estimate of the proportion of HIVST was 69.97% (95% CI 51.19%-80.25%). Subgroup analysis revealed that the proportion of actual oral mucosal fluid–based HIVST was 71.6% (95% CI 52.97%-84.95%). The proportion of actual blood-based HIVST was 67.06% (95% CI 57.69%-78.82%). Heterogeneity among the studies was found to be statistically significant (*I*^2^=98%; *P*<.001; Figure 3). To analyze the source of heterogeneity, we conducted a meta-regression to evaluate the impact that the categories of HIVST kits had on the substantial heterogeneity between the studies reporting actual HIVST uptake. However, we found no statistically significant difference in uptake of HIVST using either oral mucosal fluid– or blood-based HIVST kits (*P*=.45).

### Self-testing as Lifetime First HIV Screening

**Figure 4:**
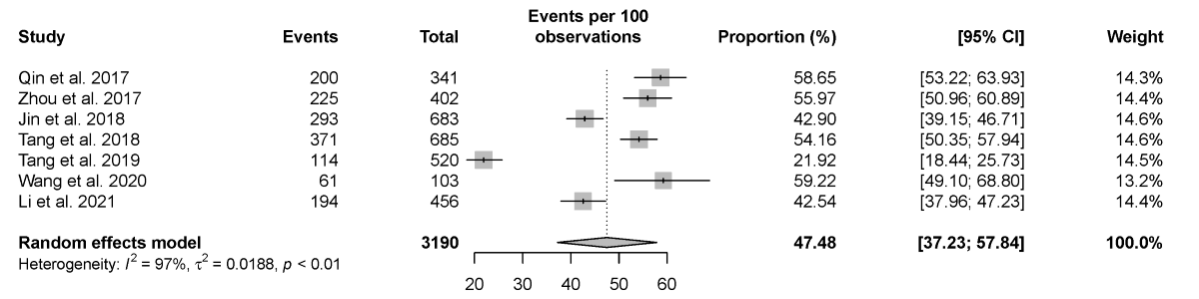
Pooled proportion of individuals using HIV self-testing (HIVST) as their first ever HIV test. Forest plot shows the proportion of individuals who tested for HIV for the first time via HIVST.

In all, 7 studies examined individuals who used HIVST as their first-ever HIV-screening experience. The pooled proportion of individuals using HIVST as their lifetime first HIV screening was 47.48% (95% CI 37.23%-57.84%) but had high heterogeneity (*I*^2^=97%; *P*<.001; Figure 4).

### Results Feedback

**Figure 5:**
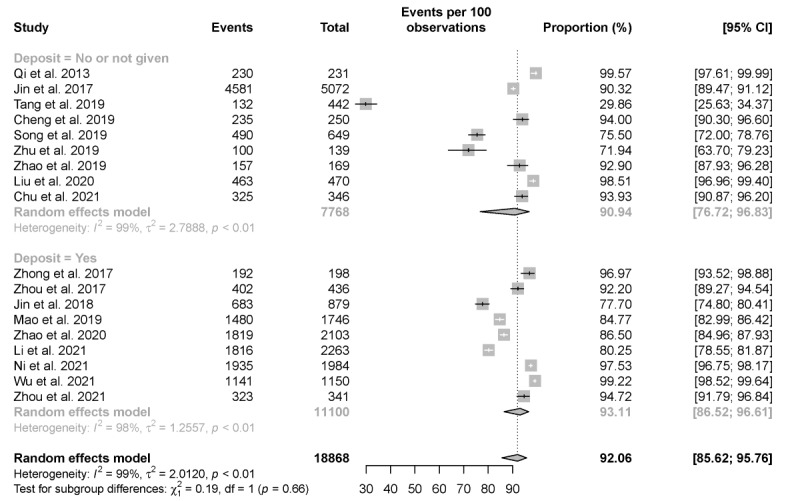
Pooled proportion of results feedback in those who self-tested for HIV. Forest plot shows estimated feedback rate among those self-tested for HIV.

Figure 5 presents the proportion of results feedback among those self-tested for HIV among 18 studies. The overall estimate of the feedback rate was 92.1% (95% CI 85.6%-95.8%) among those who self-tested for HIV. Cochran Q testing indicated substantial heterogeneity among the studies (*I*^2^=99%; *P*<.001; Figure 5).

### Reactive Rate of HIVST

**Figure 6:**
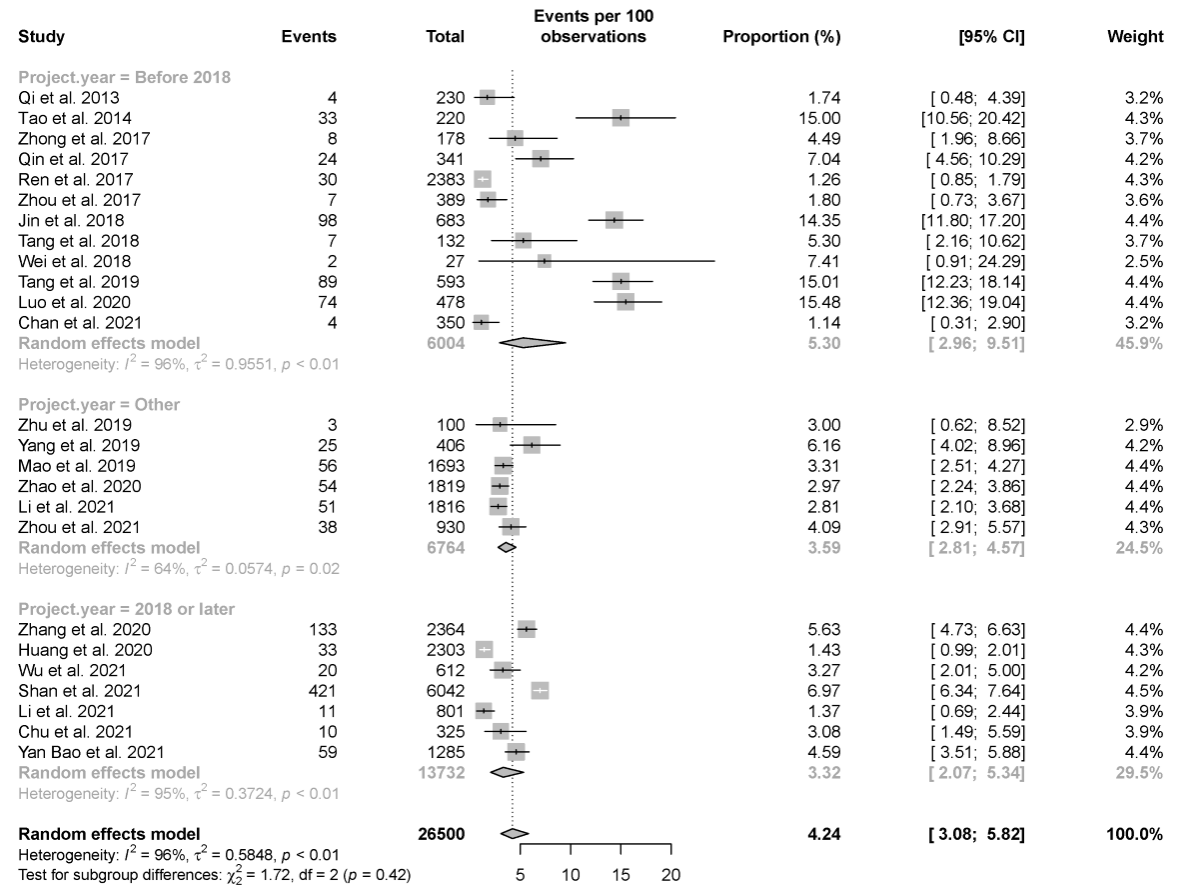
Pooled reactive rate of HIV self-testing (HIVST). Forest plot shows the reactive rate of HIVST before and after 2018. Project year "Other" refers to studies where the year that the project was conducted is unknown or cannot be distinguished.

In all, 25 studies showed the reactive rate of HIVST. The pooled reactive rate of HIVST was 4.24% (95% CI 3.08%-5.82%). Interestingly, the pooled reactive rate of HIVST before 2018 was 5.3% (95% CI 2.96%-9.51%), which is higher than that in 2018 or later (3.32%, 95% CI 2.07%-5.34%). The included studies showed high heterogeneity (*I*^2^=96%; *P*<.001; Figure 6).

### Linkage to Care

**Figure 7:**
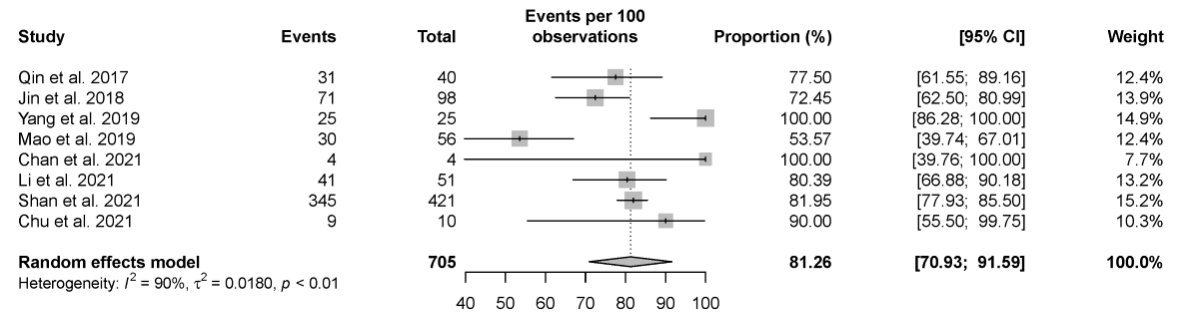
Pooled proportion of linkage to care. Forest plot shows the incidence of linkage to care among individuals whose HIV self-testing presented as reactive.

Among the 25 studies reporting the reactive rate of HIVST, 8 studies reported the incidence of linkage to care in HIV-positive self-tested individuals. The pooled proportion of linkage to care among HIV-positive individuals was 81.26% (95% CI 70.93%-91.59%; Figure 7).

### Sensitivity Analysis

Although only studies with moderate and low risk of bias were included, the pooled proportion of HIVST used previously (29.49%, 95%CI 19.99%-40%), the pooled proportion of actual uptake of HIVST (68.29%, 95% CI 51.02%-81.66%), the pooled proportion of individuals using HIVST as their lifetime first HIV screening (47.48%, 95% CI 37.23%-57.84%), the pooled feedback rate (91.38%, 95% CI 82.51%-95.97%), the pooled reactive rate of HIVST (4.21%, 95% CI 2.91%-6.09%), and the pooled proportion of linkage to care (74.68%, 95% CI 62.44%-86.92%) were similar to results obtained when including all studies (Multimedia Appendix 2).

### Publication Bias

Funnel plots for the outcomes are shown in Multimedia Appendix 3. The *P* values obtained from the Egger test for asymmetry of the funnel plot were not significant for the pooled proportion of HIVST used previously (*P*=.59), the pooled proportion of actual uptake of HIVST (*P*=.15), the pooled proportion of individuals using HIVST as their lifetime first HIV screening (*P*=.56), the pooled feedback rate (*P*=.28), and the pooled proportion of linkage to care (*P*=.58). Using the Egger test, a significant publication bias was observed in studies concerning the reactive rate of HIVST (*P*=.003), which is concerning since this tends to undermine, to a degree, the validity of the method that was used to measure the reactive rate of HIVST.

## Discussion

### Principal Findings

HIV testing is a key entry point for HIV prevention and treatment programs. Previous studies have shown that HIVST may enhance HIV testing uptake, increase HIV testing frequency, and limit potential harm among key populations [9,43]. According to Figueroa et al [65], when using HIV rapid diagnostic tests with high accuracy, those who self-test may obtain results similar to those obtained by health care workers in health care settings.

The potential factors influencing the use of HIVST include the demographic characteristics of population, age, level of education, and marital status [16,66,67]. In China, only 8% of female sex workers had ever tested for HIV using self-test kits [43]. HIVST uptake among MSM is much more prevalent than in other populations [15,31]. The higher uptake of HIVST in MSM may be attributable to HIV awareness raising by MSM-associated social organizations. For instance, crowdsourcing has been an effective strategy for enhancing HIVST uptake in MSM, especially in low- and middle-income countries [29]. Furthermore, the rate of HIVST use among MSM who use pre-exposure prophylaxis (PrEP), especially among those on daily PrEP, was observed to be high in a multicenter trial in China [68]. Thus, more extensive and specific applications of HIVST, such as using HIVST during the follow-up of PrEP users, may generate improved data for HIVST uptake and use. All forms of sex work are illegal in China, which restricts female sex workers and their male clients from actively seeking relevant and valuable knowledge regarding HIV. Therefore, inventive strategies are required to enhance the uptake of HIVST in key populations who are at high risk of HIV infection but possess limited knowledge of HIV prevention. In the 23 studies reporting the proportion of those who previously self-tested, only 2 studies [14,23] reported on the number of oral fluid–based HIVST used by self-testers (13.87% and 29%, respectively). Although it has been reported in the past that oral fluid–based HIVST is valid, acceptable, and accurate [5], the practical application of this testing method remains a challenge in China. Notably, oral fluid–based HIVST kit users in China were more likely to make errors during the oral HIVST testing procedure. Data gleaned from statistical meta-regression suggest that the category of the testing kit may not be the main or only reason for the actual difference in uptake between oral and blood-based HIVST kits.

According to our meta-analysis, it is estimated that approximately half (47.48%) of all individuals who were tested for HIV in China used HIVST as their first-ever test. HIVST has the potential to reach high-risk populations who have never been tested for HIV or who refuse voluntary counseling and testing. Qin et al [23], in contrast, observed that MSM who find sexual partners through the internet prefer medical facility–based testing rather than self-testing as their first-ever HIV test. This finding might be attributable to the fact that MSM who use the internet may have legitimate concerns regarding the accuracy of self-test kits after coming across relevant information on the internet, given their regular and routine access to internet-based information, in this instance, related to the overall quality and validity of HIVST kits on the internet [23]. HIVST with digital support may be an effective way to engage with those who self-test for HIV for the first time and some hard-to-reach populations, according to McGuire et al [69]. HIVST along with web-based counseling may be an effective strategy to increase the prevalence of HIV testing and reduce sexual risk behaviors [70], especially during the COVID-19 pandemic, when accessing HIV testing may have structural, regulatory, and psychological public health–related barriers [71,72]. Therefore, raising awareness of the accuracy and reliability of HIVST in a web-based digital manner might be desirable. In addition, monetary incentives combined with peer referral in the MSM population is also an effective manner to encourage first-time testing [73]. However, it is essential that other novel and inventive approaches for the promotion of first-time HIV testing are further explored.

The overall result feedback rate in those who self-test for HIV is known to be relatively high. Jin et al [37] compared the behaviors between those who did and those who did not submit their results after self-testing and observed, curiously, that individuals at lower HIV risk were more reluctant to submit their test results [37]. The lower feedback rate among individuals at lower HIV risk may lead to an inaccurate estimation of HIV prevalence in these populations. In this meta-analysis, we found that the cost of participants acquiring self-testing kits did not play a significant role in influencing results feedback.

The pooled reactive rate of HIVST among MSM in our study is similar to the overall national prevalence of HIV among MSM from 2001 to 2018, as estimated by Dong et al [74] (4.6%, 95% CI 3.2%-6.5% vs 5.7%, 95% CI 5.4%-6.1%). One recent study suggested that the lower reactive rate of HIVST is likely to be associated with a wider participant base in the study, and participants are not necessarily restricted to potential high-risk individuals only [3]. Our findings support the aforementioned speculation—that the reactive rate in China has declined steadily along with the widespread use of HIVST after 2018. However, beyond that, the reduced reactive rate may also reflect a demonstration of the significance of HIVST for HIV prevention.

Choko et al [75] indicated that financial incentives and partner-delivered approaches may likely increase male linkage into posttest HIV care. Among the studies providing self-test kits (excluding 2 studies with small sample size [48,60]; n≤10), those providing self-test kits distributed by sexual partners [53] showed a higher linkage-to-care rate among HIV-positive patients (81.9%), whereas for those studies only distributing testing kits by post [37,40], the linkage-to-care rate was from 53.6% to 72.4%. Our review observed that sexual partners may play a critical role in accessing timely medical intervention for their partners with HIV-positive self-tests.

### Limitations

We acknowledge several limitations to our study. First, most of the data used in our study were derived from the MSM population, and our analysis is based exclusively on this data. Therefore, the results of our study provide only limited outcomes and knowledge with respect to the application of HIVST in other high-risk populations. Second, data associated with the use of HIVST over extended periods are limited. One recent longitudinal study observed that HIVST adherence reduced to a paltry 10% during 1-year of follow-up [76]. Thus, the potential role of HIVST use over prolonged periods requires further exploration. Third, HIVST kits provided by research sponsors were available for free or on a refundable basis in the included studies. The effect of actual cost of access to HIVST kits in the real world in China was, therefore, not assessed or commented upon in our discussion.

### Conclusion

In summary, HIVST has evolved over recent years into an important pillar of HIV prevention in China. However, the use of HIVST in non-MSM populations requires sustained upscaling. The long-term applications of HIVST and the effects of self-financing of HIVST in China have yet to be explored.

## Appendix

Multimedia Appendix 1 Search strategy.

Multimedia Appendix 2 Sensitivity Analysis.

Multimedia Appendix 3 Funnel plots for the outcomes.

## Abbreviations

HIVST: HIV self-testing
MSM: Men who have sex with men
PrEP: pre-exposure prophylaxis
PRISMA: Preferred Reporting Items for Systematic Reviews and Meta-Analyses
