# Supplementary material for: The Practice and Potential Role of HIV Self-testing in China: Systematic Review and Meta-analysis
Source: JMIR Public Health Surveill. 2022 Dec 2;8(12):e41125. doi: 10.2196/41125 (PMC9758640; doi:10.2196/41125)
Supplement: Multimedia Appendix 1 [file publichealth_v8i12e41125_app1.docx]

**Multimedia Appendix 1**

**Search strategy**

**1. PUBMED**

#1 “HIV Infections” [MeSH] OR “HIV” [MeSH] OR “hiv” [tw] OR “hiv-1” [tw] OR “hiv-2” [tw] OR “hiv1” [tw] OR “hiv2” [tw] OR hiv infect* [tw] OR “human immunodeficiency virus” [tw] OR “human immunedeficiency virus” [tw] OR “human immuno-deficiency virus” [tw] OR “human immune-deficiency virus” [tw] OR ((human immun*) AND (“deficiency virus” [tw])) OR “acquired immunodeficiency syndrome” [tw] OR “acquired immunedeficiency syndrome” [tw] OR “acquired immuno-deficiency syndrome” [tw] OR “acquired immune-deficiency syndrome” [tw] OR ((acquired immun*) AND (“deficiency syndrome” [tw])) OR "Sexually Transmitted Diseases, Viral" [MeSH:NoExp]

#2 “hiv self-testing” [All Fields] OR “hiv self-test” [All Fields] OR "hivst" [All Fields] OR home test*[tiab] OR rapid test*[tiab] OR home self test*[tiab] OR home-based self test*[tiab] OR "self test"[tiab] OR self testing [tiab] OR “home test” [tiab] OR home testing [tiab]

#3 “China” [tw] OR “Chinese” [tw] OR “People's Republic of China” [tw] OR “Mainland China” [tw]

#4 "Animals"[Mesh] NOT ("Animals"[Mesh] AND "Humans"[Mesh])

#5 (#3 AND #2 AND #1) NOT #4

**2. EMBASE**
#1 'human immunodeficiency virus infection'/exp OR 'human immunodeficiency virus'/exp OR hiv:ti OR hiv:ab OR 'hiv-1':ti OR 'hiv-1':ab OR 'hiv-2':ti OR 'hiv-2':ab OR 'human immunodeficiency virus':ti OR 'human immuno deficiency':ab OR 'human immuno-deficiency virus':ti OR 'human immuno-deficiency virus':ab OR 'human immunedeficiency virus':ti OR 'human immune deficiency virus':ab OR 'human immune- deficiency virus':ti OR 'human immune-deficiency virus':ab OR 'acquired immune-deficiency syndrome':ti OR 'acquired immune-deficiency syndrome':ab OR 'acquired immunedeficiency syndrome':ti OR 'acquired immunedeficiency syndrome':ab OR 'acquired immunodeficiency syndrome':ti OR 'acquired immunodeficiency syndrome':ab OR 'acquired immuno-deficiency syndrome':ti OR 'acquired immuno-deficiency syndrome':ab AND [humans]/lim

#2 'self evaluation'/exp OR (‘hivst’ OR ‘hiv self-testing’ OR ‘hiv self-test’ OR ‘hiv home testing’ OR ‘hiv home test’ OR ‘hiv rapid test’ OR ‘hiv rapid testing’ OR ‘home self test’ OR ‘home self testing’ OR ‘home-based self test’ OR ‘home-based self testing’):ab,ti,de,ca AND [humans]/lim

#3 ‘China’/exp OR (‘Chinese’ OR ‘Chinese People*s Republic’ OR ‘People*s Republic of China’ OR ‘mainland China’):ab,ti,ca

#1 AND #2 AND #3

**3. Web of science**

#1 TS=(HIV OR HIV-1 OR HIV-2 OR acquired immunodeficiency syndrome) OR TI=("acquired immunodeficiency syndrome" OR "AIDS" OR "human immunodeficiency virus" OR "HIV") OR AB=("acquired immunodeficiency syndrome" OR "AIDS" OR "human immunodeficiency virus" OR "HIV")

#2 TS=(HIV self-testing OR HIV self-test OR HIVST OR HIV home test OR HIV rapid testing) OR TI=(“home test*” OR “rapid test*” OR “home self-test*” OR “home-based self test*” OR "self test" OR “self-testing” OR “home test” OR “home testing”) OR AB=(“home test*” OR “rapid test*” OR “home self-test*” OR “home-based self test*” OR "self test" OR “self-testing” OR “home test” OR “home testing”)

#3 TI=(“China” OR “Chinese” OR “People's Republic of China” OR “Mainland China”) OR AB=(“China” OR “Chinese” OR “People's Republic of China” OR “Mainland China”)

#1 AND #2 AND #3

**4. CNKI（****in Chinese）**

#1 SU=(艾滋病 OR 人类免疫缺陷病毒 OR 获得性免疫缺陷综合征 OR HIV OR AIDS) OR TI=(艾滋病 OR 人类免疫缺陷病毒 OR 获得性免疫缺陷综合征 OR HIV OR AIDS) OR AB=(艾滋病 OR 人类免疫缺陷病毒 OR 获得性免疫缺陷综合征 OR HIV OR AIDS)

#2 SU=(HIV自检 OR 艾滋病自检) OR TI=(自检 OR 自我检测 OR 快速检测 OR 快检 OR 自我快速检测 OR 抗体自检 OR 抗体自我检测) OR AB=(自检 OR 自我检测 OR 快速检测 OR 快检 OR 自我快速检测 OR 抗体自检 OR 抗体自我检测)

#1 AND #2

**5. Wanfang（in Chinese）**

#1 (主题:("艾滋病" OR "人类免疫缺陷病毒" OR "获得性免疫缺陷综合征" OR "HIV" OR "AIDS") OR 题名或关键词:("艾滋病" OR "人类免疫缺陷病毒" OR "获得性免疫缺陷综合征" OR "HIV" OR "AIDS") OR 摘要:("艾滋病" OR "人类免疫缺陷病毒" OR "获得性免疫缺陷综合征" OR HIV OR AIDS)) AND (主题:("HIV自检" OR "艾滋病自检") OR 题名或关键词:("自检" OR "自我检测" OR "自我快速检测" OR "抗体自检" OR "抗体自我检测") OR 摘要:("自检" OR "自我检测" OR "自我快速检测" OR "抗体自检" OR "抗体自我检测"))
