# Supplementary material for: The Practice and Potential Role of HIV Self-testing in China: Systematic Review and Meta-analysis
Source: JMIR Public Health Surveill. 2022 Dec 2;8(12):e41125. doi: 10.2196/41125 (PMC9758640; doi:10.2196/41125)
Supplement: Multimedia Appendix 2 [file publichealth_v8i12e41125_app2.docx]

**Multimedia Appendix 2. Sensitivity Analysis.**


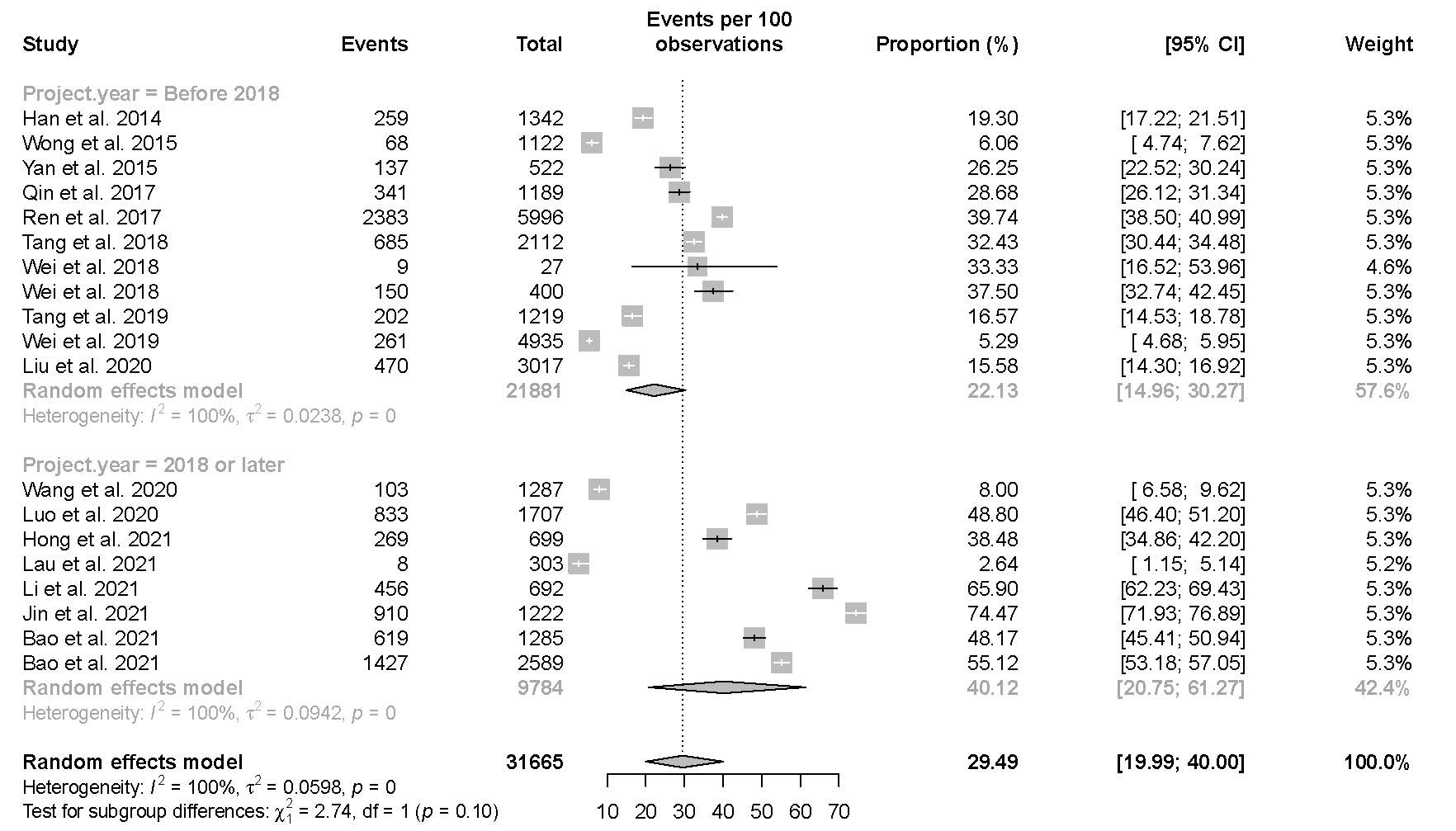


Figure S1. Sensitivity analysis for studies with low and moderate risk of bias on proportion of HIVST used previously.


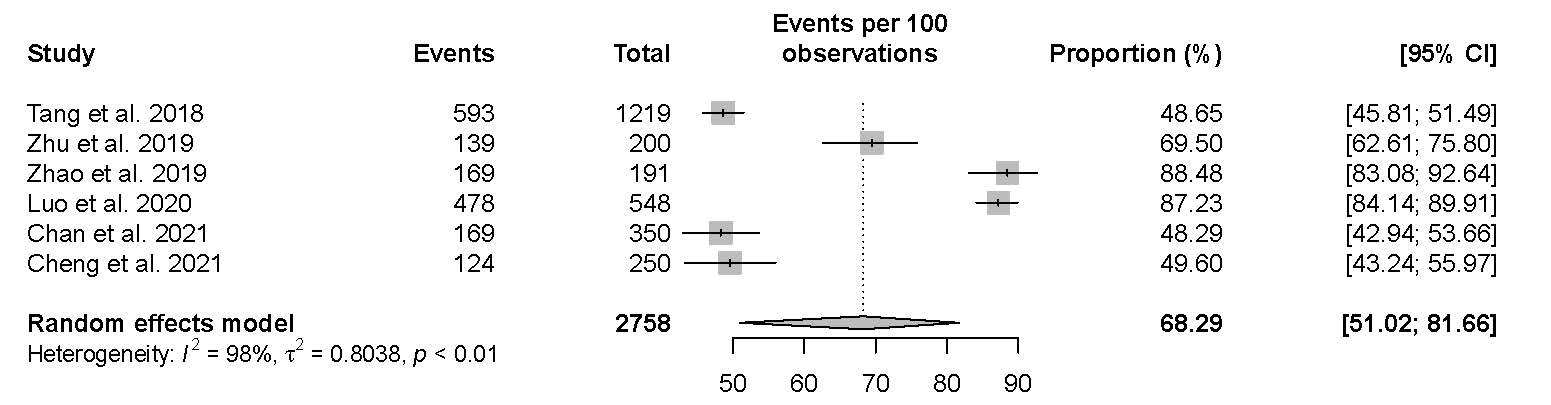


Figure S2. Sensitivity analysis for studies with low and moderate risk of bias on proportion of actual HIVST uptake.


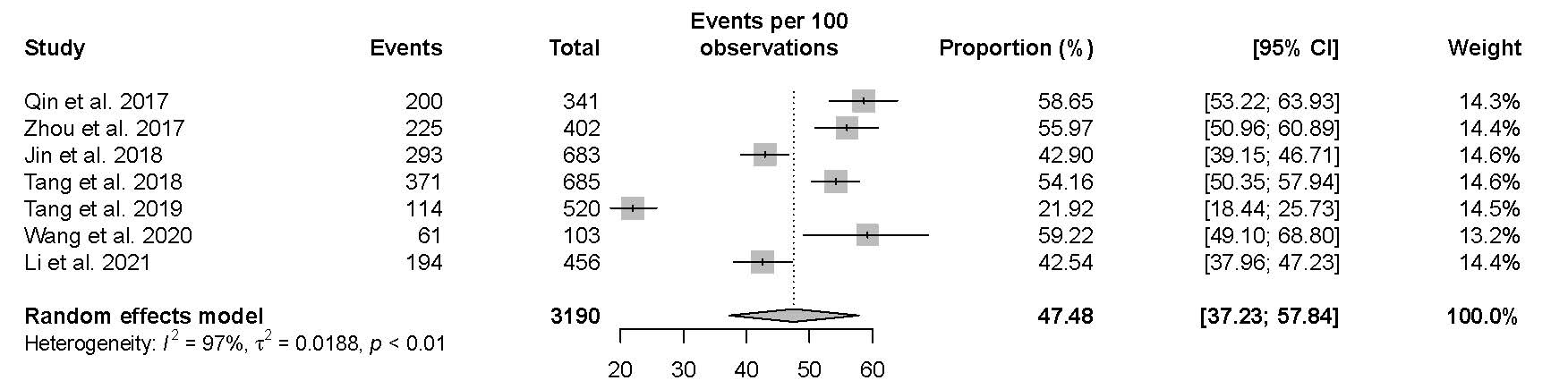
Figure S3. Sensitivity analysis for studies with low and moderate risk of bias on pooled proportion of individuals using HIVST as their first ever HIV test.


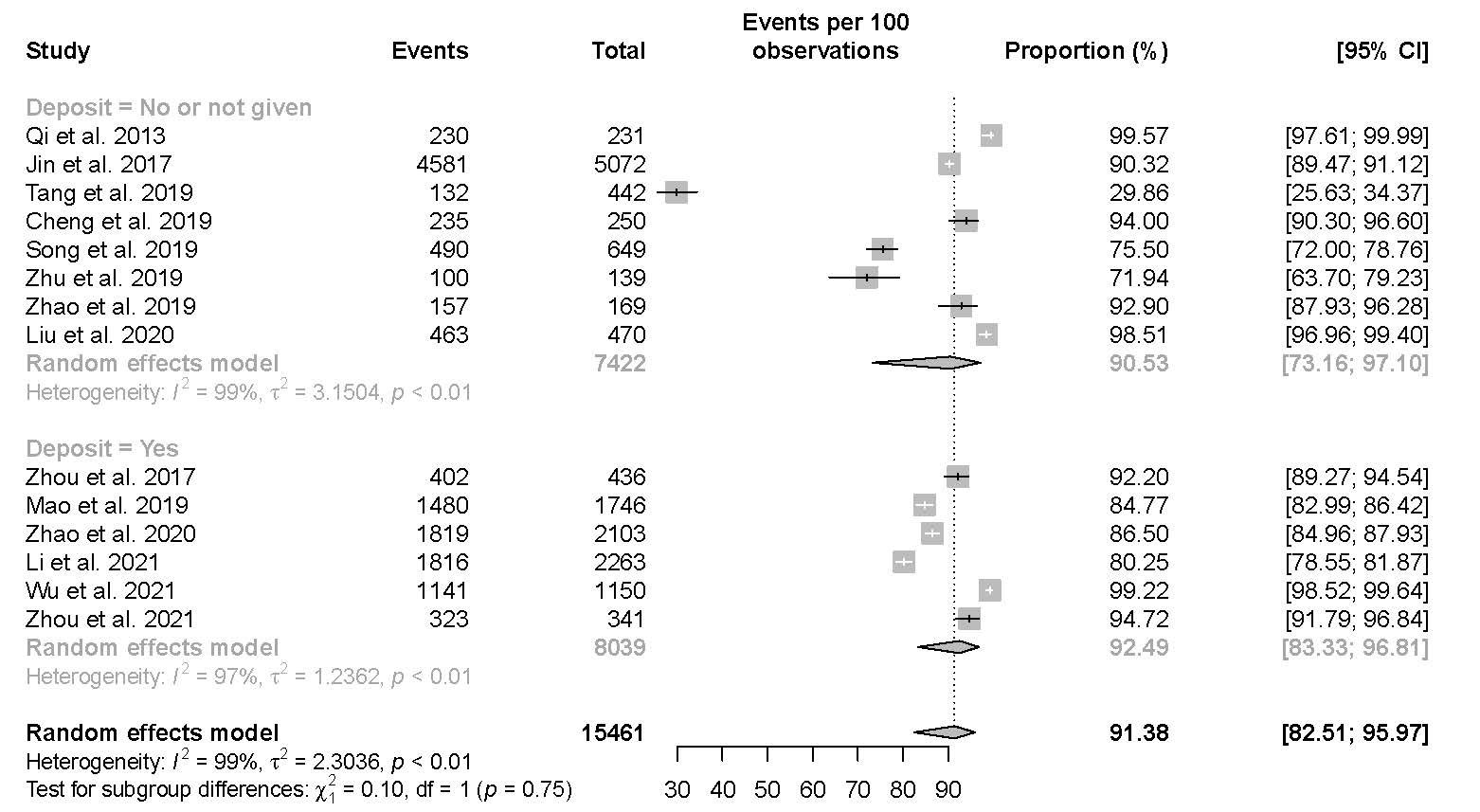


Figure S4. Sensitivity analysis for studies with low and moderate risk of bias on proportion of results feedback individuals in those self-tested for HIV.


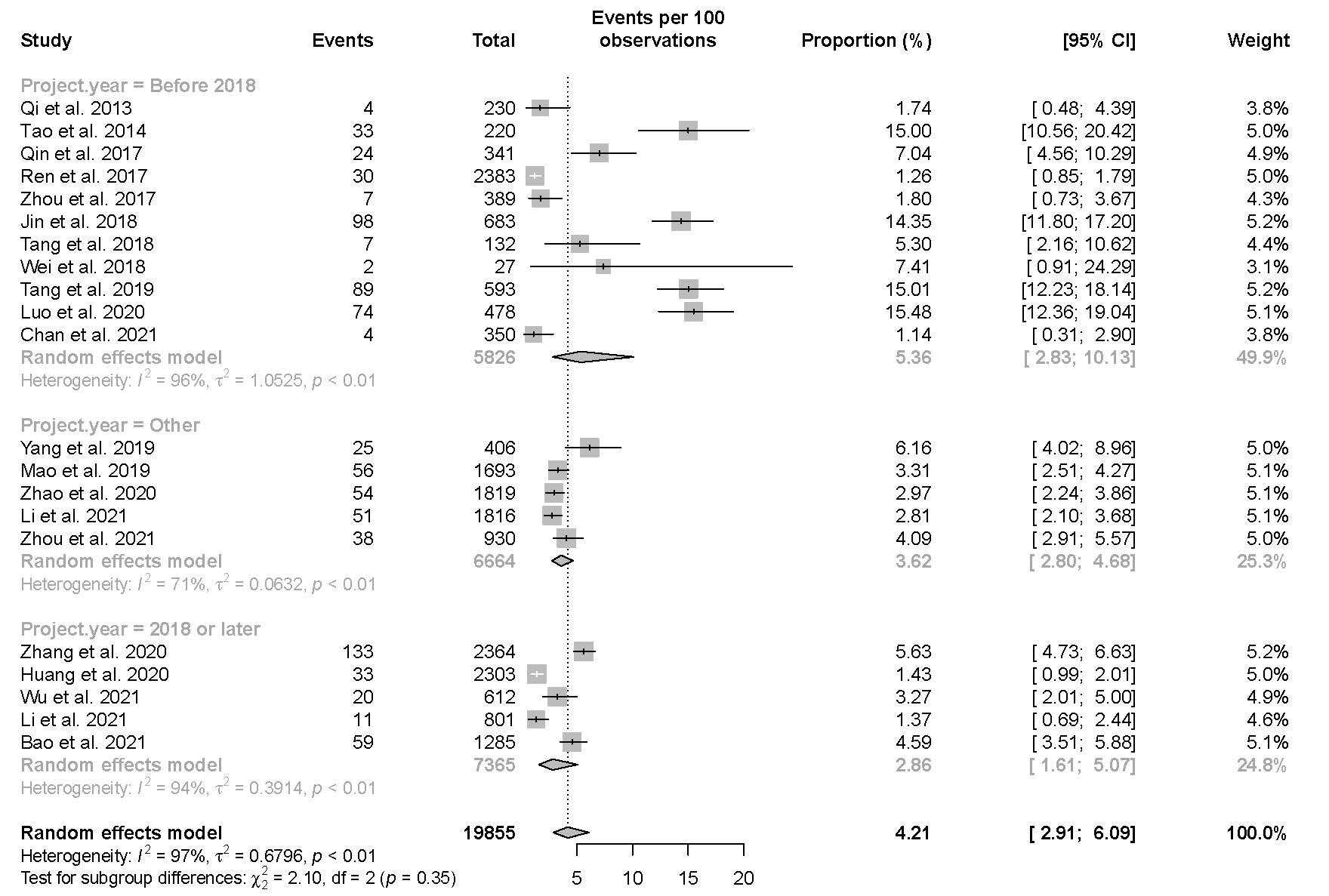


Figure S5. Sensitivity analysis for studies with low and moderate risk of bias on reactive rate of HIVST.


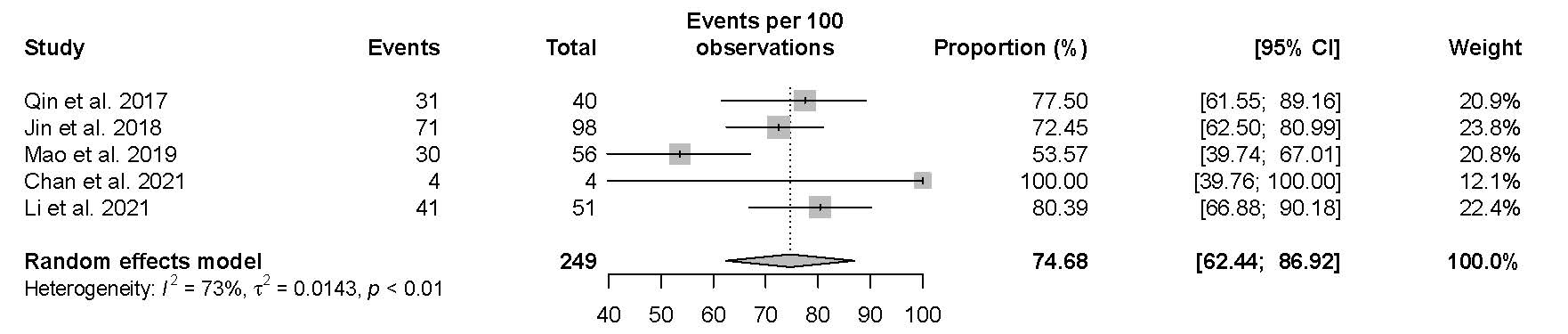


Figure S6. Sensitivity analysis for studies with low and moderate risk of bias on proportion of linkage to care.
