# Supplementary material for: The Practice and Potential Role of HIV Self-testing in China: Systematic Review and Meta-analysis
Source: JMIR Public Health Surveill. 2022 Dec 2;8(12):e41125. doi: 10.2196/41125 (PMC9758640; doi:10.2196/41125)
Supplement: Multimedia Appendix 3 [file publichealth_v8i12e41125_app3.docx]

**Multimedia Appendix 3. Funnel plots for the outcomes.
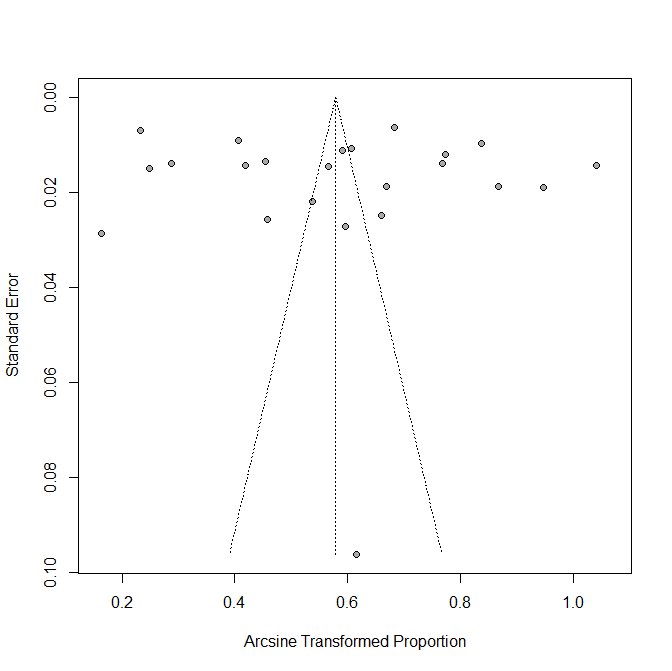
**

Figure S1. Funnel plot for studies on proportion of HIVST used previously.


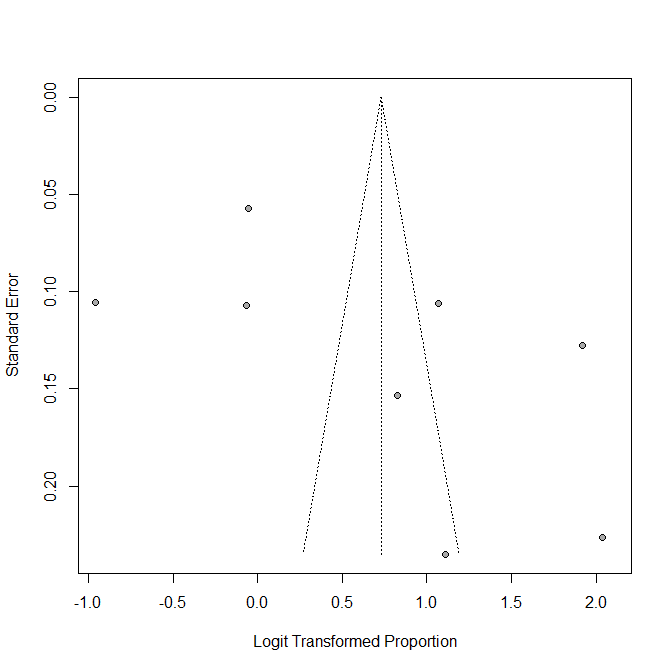


Figure S2. Funnel plot for studies on proportion of actual HIVST uptake.


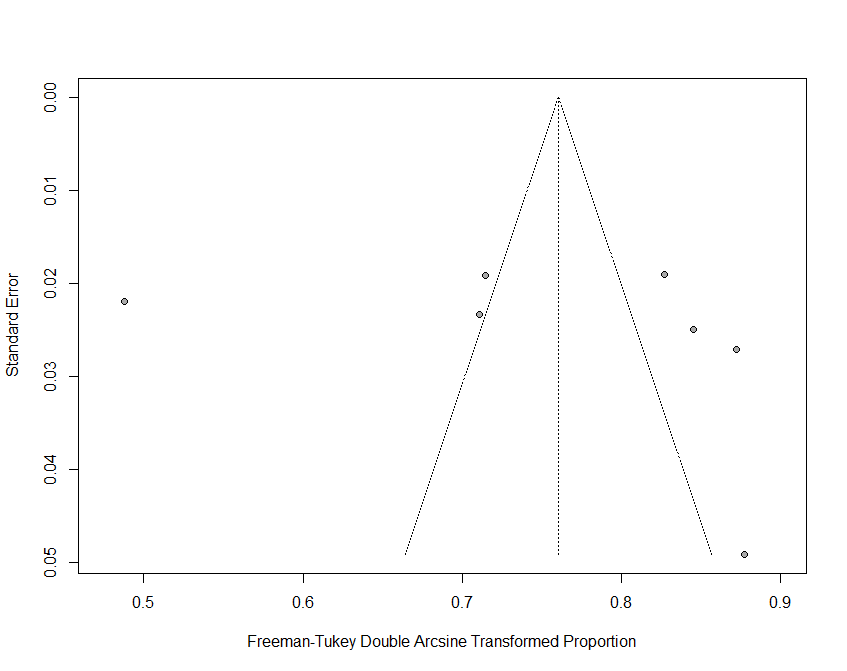
Figure S3. Funnel plot for studies on proportion of individuals using HIVST as their first-ever HIV test.


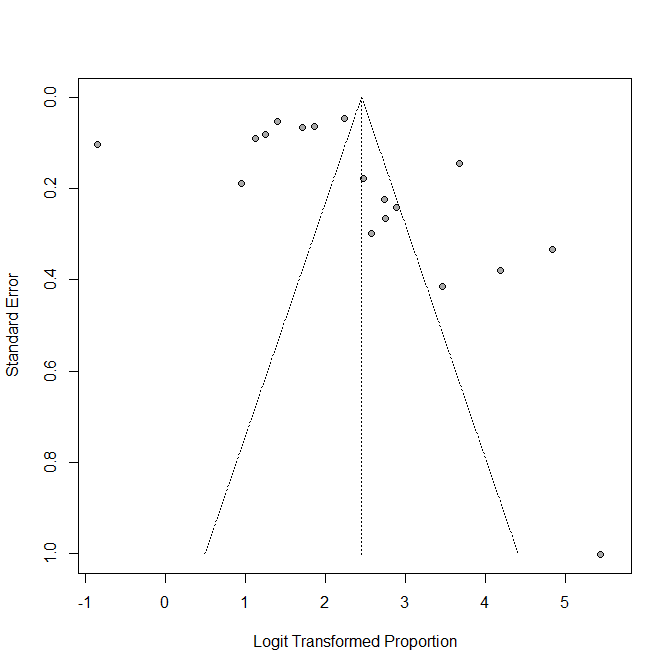


Figure S4. Funnel plot for studies on proportion of results-feedback individuals in those self-tested for HIV.


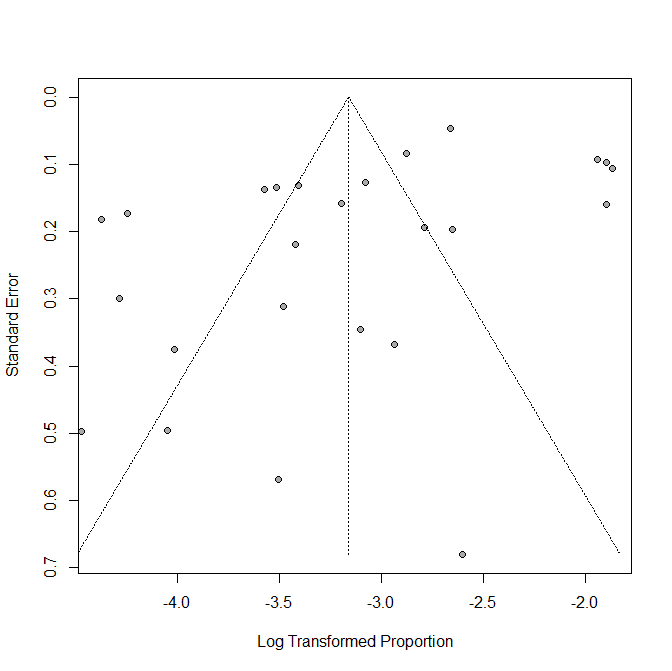


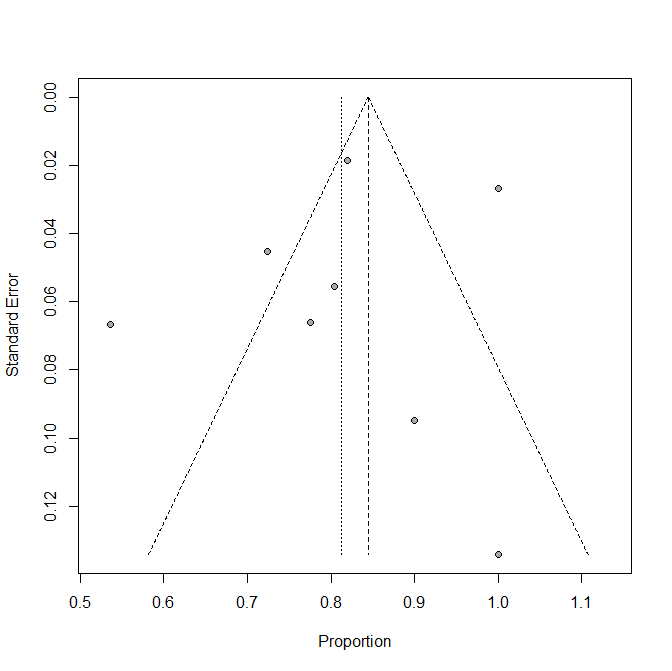
Figure S5. Funnel plot for studies on reactive rate of HIVST.

Figure S6. Funnel plot for studies on proportion of linkage to care.
